# Functional Interference due to Pain Following Palliative Radiotherapy for Bone Metastases Among Patients in Their Last Three Months of Life

**DOI:** 10.4021/wjon290w

**Published:** 2011-04-09

**Authors:** Kristopher Dennis, Liying Zhang, Lori Holden, Florencia Jon, Elizabeth Barnes, May Tsao, Cyril Danjoux, Arjun Sahgal, Liang Zeng, Kaitlin Koo, Luluel Khan, Amanda Caissie, Edward Chow

**Affiliations:** a Rapid Response Radiotherapy Program, Department of Radiation Oncology, Odette Cancer Center, Sunnybrook Health Sciences Center, University of Toronto, Toronto, Ontario, Canada

**Keywords:** Bone metastases, Pain, Palliative care, Prognosis, Radiotherapy, Quality of life

## Abstract

**Background:**

To compare the self-reported ratings of functional interference caused by pain between patients who did and did not respond to palliative radiotherapy for bone metastases during their last three months of life.

**Methods:**

A prospectively gathered Brief Pain Inventory (BPI) database compiled from patients receiving palliative radiotherapy for painful bone metastases was reviewed. Demographic and clinical data, pain response rates and self-reported ratings of functional interference caused by pain were analyzed for those patients who died within three months of beginning radiotherapy.

**Results:**

From 400 patients in the database, 83 died within 3 months of beginning radiotherapy. There were 54 male and 29 female patients. Their median age was 69 years and their median KPS was 70. The three most common primary cancers were lung (40%), gastrointestinal (16%) and breast (14%). For patients with available follow-up information the 1-month overall pain response rate was 78% and the 2-month rate was 83%, which include both complete and partial responses as defined by the International Bone Metastases Consensus. At 1 month, patients responding to treatment reported significantly less interference by pain on their general activity, walking ability, normal work, sleeping, and enjoyment of life than did patients not responding to treatment.

**Conclusions:**

Patients that responded to treatment reported less functional interference due to pain than did patients who did not respond. Despite being very near the end of life, patients responding to palliative radiotherapy for painful bone metastases may benefit from more than pain relief alone.

## Introduction

Bone metastases cause significant pain and functional disability as a result. They are effectively palliated with external beam radiotherapy [1, 2]; however, the latency to response is variable. Although the median time from treatment to some degree of pain relief is believed to lie between 1 and 4 weeks [2, 3], neither the timing nor the magnitude of this relief have been clearly demonstrated. This is problematic for patients nearing the end of life who require treatments that will return them to an optimal functional level in the shortest period of time with the fewest treatment-related side effects [4, 5].

There is very limited data on the efficacy of radiotherapy for palliating pain from bone metastases among patients nearing the end of life [6], and no data on the impact of treatment on the functional abilities of patients in this setting. The present study addresses this problem by examining pain relief and functional abilities following radiotherapy in patients with short-lived survival.

## Materials and Methods

### The brief pain inventory

The Brief Pain Inventory (BPI) is a pain assessment questionnaire that was designed for use with patients with cancer [7-9]. It measures both pain severity and the interference pain causes on different functional domains using 11-point ordinal rating scales. Pain is rated from 0 = ‘no pain’ to 10 = ‘pain as bad as you can imagine’. The interference of pain on functional domains is rated from 0 = ‘does not interfere’ to 10 = ‘completely interferes’. The functional domains measured are general activity, mood, walking ability, normal work (including housework and work outside the home), relations with other people, sleeping, and enjoyment of life. The BPI has been validated in populations of patients with advanced cancer and typically requires less than 5 minutes to complete [10].

### Study design

The study population was selected from a cohort of patients that received palliative radiotherapy for bone metastases between May 2003 and November 2007 at the Rapid Response Radiotherapy Program (RRRP) at the Odette Cancer Center, Sunnybrook Health Sciences Center, Toronto, Ontario, Canada. All data was extracted from a prospectively gathered database that contained oncologic demographic data including dates of death, as well as BPI questionnaire responses. Data from patients dying within three months of the first day of radiotherapy was selected for analysis. All patients completed baseline BPI questionnaires in person on the day of initial assessment, and trained clinical research assistants attempted to have all patients complete follow-up BPI questionnaires over the telephone at 1 and 2 months following the end of radiotherapy. Data from 1 week prior to and 1 week following the 1 and 2 month follow-up time points respectively was permitted. Patients with data from both the baseline and the 1 and/or 2 month follow-up time points formed the ‘study population’ for all analyses, while those patients with baseline data only formed the ‘lost to follow-up population’. Other data analyzed included age, gender, Karnofsky Performance Status (KPS), the primary cancer site, the site of irradiated bone metastases, and the fractionation schedule employed (single or multiple). Analgesic consumption for the 24 hour period preceding the time of assessment was also recorded each time the BPI was administered, and opioid consumption was converted to a daily total oral morphine equivalent. Approval for this study was obtained from the research ethics board of the Sunnybrook Health Sciences Center.

Response to radiotherapy was defined according to the endpoints of the International Bone Metastases Consensus [11]. A complete response (CR) was a pain score of zero at the irradiated site with no concomitant increase in analgesic intake. A partial response (PR) was either A) a 2-point reduction in pain score at the irradiated site and no concomitant increase in analgesic intake or B) a 25% decrease in analgesic intake with no concomitant increase in pain score at the irradiated site. Patients with responses to therapy not considered a CR or PR were deemed non-responders.

### Statistical analysis

The Statistical Analysis Software (SAS version 9.2 for Windows) was used for all analyses. Descriptive statistics summarized demographic data. The Fisher exact test compared categorical demographic variables between the study population and the lost to follow-up population, while the Wilcoxon rank-sum non-parametric test compared continuous variables between those groups. The Wilcoxon rank-sum non-parametric test also compared BPI functional interference scores between responders and non-responders. Two-sided P-values of < 0.05 were considered statistically significant for all analyses.

## Results

The BPI database contained 400 patients in total, and 83 of them had died within 3 months of beginning radiotherapy. There were 54 males and 29 females. Their median age was 69 years and their median KPS was 70. The three most common primary cancers were lung (40%), gastrointestinal (16%) and breast (14%). Sixty-nine percent of these patients received single fraction therapy. Both baseline and follow-up data were available for 42% of the cohort (n = 35), and these patients constituted the study population. The remaining 48 patients constituted the lost to follow-up population. The baseline demographic and clinical characteristics of these cohorts are displayed in Table 1. The study population baseline pain score (mean ± SD) was 8.0 ± 1.96, its baseline daily total oral morphine equivalent (mean ± SD) was 111.2 mg ± 172.4 mg, and its baseline BPI functional interference scores (mean ± SD) were: general activity (7.34 ± 2.99), mood (5.17 ± 3.36), walking ability (6.29 ± 3.53), normal work (7.49 ± 3.22), relations with other people (4.37 ± 3.51), sleeping (4.49 ± 3.74), and enjoyment of life (8.09 ± 2.72). Seventy-seven percent of patients in the study population began treatment on the day of their baseline assessment. The median latency to treatment for the remaining 23% of patients was 4 days (range 1 - 11 days). The median survival of the study population was 64 days from the first day of radiotherapy, while that for patients lost to follow-up was 36 days.

**Table 1:** Demographic and Clinical Characteristics of the 83 Patients Dying Within 3 Months of Beginning Radiotherapy, as Divided Between the Study Population and Patients Lost to Follow-up

	Study Population (n = 35)	Lost to Follow-up (n = 48)
Age (years)		
Median (Range)	68 (42 - 85)	70 (41 - 86)
Gender		
Male	20 (57.1%)	34 (70.8%)
Female	15 (42.9%)	14 (29.2%)
KPS		
Median (Range)	70 (30 - 90)	60 (40 - 90)
Primary Tumor Site		
Lung	11 (31.4%)	22 (45.8%)
Gastrointestinal	8 (22.9%)	5 (10.4%)
Breast	8 (22.9%)	4 (8.3%)
Prostate	5 (14.3%)	5 (10.4%)
GU (non-Prostate)	3 (8.6%)	3 (6.3%)
Primary Unknown	0	5 (10.4%)
Others	0	4 (8.3%)
Irradiated Site		
Spine	11 (31.4%)	19 (39.6%)
Sacrum/Pelvis	11 (31.4%)	4 (8.3%)
Lower Extremity	7 (20%)	12 (25%)
Upper Extremity	2 (5.7%)	4 (8.3%)
Others	4 (11.4%)	9 (18.8%)
Fractionation		
Single	27 (77.1%)	30 (62.5%)
Multiple	8 (22.9%)	18 (37.5%)

### Comparison of baseline characteristics between the study population and patients lost to follow-up

Comparisons between the study population and patients lost to follow-up with respect to baseline demographics, clinical information and BPI functional domain ratings are listed in Table 2. The only significant difference found was that patients within the study population were more likely to have had a pelvic/sacral site of irradiated bone metastases (P = 0.0095).

**Table 2:** P-Values From Comparisons Between the Study Population and Patients Lost to Follow-up With Respect to Baseline Demographics, Clinical Information and BPI Functional Domain Ratings

Age	0.40
Gender	0.25
KPS	0.13
Primary Tumor Site	NS
Irradiated Site	
Spine	0.49
Lower Extremity	0.79
Sacrum/Pelvis	0.0095*
Upper Extremity	0.65
Other	0.54
Fractionation	NS
Functional Domain	
General Activity	0.95
Mood	0.31
Walking Ability	0.76
Normal Work	0.37
Relations with Other People	0.54
Sleeping	0.45
Enjoyment of Life	0.36

*denotes a significant difference, NS denotes non-significance.

### Study population response rates

Ninety-one percent of patients in the study population were available for the 1 month time point analysis (n = 32), while only 17% were available for the 2 month time point (n = 6). The 1 month overall response rate for available patients was 78% (0% complete responder and 78% partial responders) while the 2 month overall response rate for available patients was 83% (0% complete responder and 83% partial responders).

### Comparison of functional domain ratings between responders and non-responders within the study population

The BPI functional interference scores at 1-month following radiotherapy for responders and non-responders respectively (mean ± SD) were: general activity (4.00 ± 4.07 for responders vs. 9.00 ± 1.91 for non-responders), mood (3.29 ± 3.87 vs. 5.50 ± 4.37), walking ability (3.71 ± 4.16 vs. 7.14 ± 3.08), normal work (3.95 ± 4.36 vs. 8.57 ± 3.78), relations with other people (2.57 ± 3.72 vs. 5.00 ± 4.38), sleeping (2.45 ± 3.28 vs. 6.67 ± 3.88), and enjoyment of life (4.62 ± 4.33 vs. 8.67 ± 2.16). P-values from comparisons between the responder and non-responder ratings at 1 and 2 months are listed in Table 3. At 1 month, responders indicated that their pain was causing significantly less interference on a number of functional domains compared to non-responders: general activity (P = 0.0047), walking ability (P = 0.046), normal work (P = 0.016), sleeping (0.021) and enjoyment of life (0.041). No significant differences between groups were found at 2 months.

**Table 3:** P-Values From Comparisons Between the BPI Functional Domain Ratings for Responders and Non-Responders Within the Study Population (N = 35) at 1 and 2 Months Following the End of Radiotherapy

	1 month (n = 32 available patients)	2 months (n = 6 available patients)
Functional Domain		
General Activity	0.0047*	0.37
Mood	0.18	NS
Walking Ability	0.046*	0.23
Normal Work	0.016*	0.37
Relations With Other People	0.24	NS
Sleeping	0.021*	NS
Enjoyment of Life	0.041*	NS

*denotes a significant difference, NS denotes non-significance.

## Discussion

This is the first study that has investigated the effect of palliative radiotherapy for bone metastases on the functional abilities of patients within their last 3 months of life. Although it seems intuitive that the patients who responded to treatment also reported significantly less functional interference due to their pain at one month, this relationship had not been formally evaluated previously in a population of patients with such a limited lifespan. The distinction is important, as patients with painful bone metastases nearing the end of life commonly suffer from comorbidities and declining performance status that could mask any functional benefit that would otherwise be seen following pain relief from radiotherapy. Wu and colleagues noted a significant correlation between pain reduction and improvements in BPI functional interference scores among 109 patients who received palliative radiotherapy for bone metastases; however, the survival times of the cohort were not reported [12]. Our group also previously reported the same correlation among 199 patients receiving palliative radiotherapy for bone metastases with varying life spans [13]. Although no significant differences in interference ratings between the groups in the present study were observed at 2 months, as very few patients contributed data for this time point, no meaningful conclusions can be drawn.

Between 1996 and 1998, 1157 patients were enrolled into the Dutch Bone Metastases Study which demonstrated that a single 8 Gy fraction and six 4 Gy fractions are equally effective at providing pain relief from uncomplicated bone metastases [14]. The outcomes of the 274 patients within this trial who died within 12 weeks of randomization were recently described by Meeuse and colleagues [6]. With an impressive 91% of patients having available follow-up data, the authors reported 47% and 44% overall response rates for patients receiving single- and multiple-fraction therapy respectively, with a median time to pain response for both groups of 2 weeks (range, 1 - 9 weeks). Response rate definitions were consistent with those of the International Bone Metastases Consensus [11]. This study did not, however, report any functional or quality of life outcomes.

Recent discussions have emphasized the need to better integrate palliative radiotherapy into the treatments commonly offered to patients nearing the end of life [15, 16]. The results from our cohort support this position by demonstrating that patients may indeed derive pain relief prior to passing away. The results also suggest that patients responding to treatment may notice improvements in their ability to perform common daily activities as a result. If single fraction therapy is properly utilized for uncomplicated bone metastases, the inconvenience treatment poses to patients and their families should also be minimal.

Some limitations of the present study warrant comment. Having follow-up data missing from over half of the study population poses a risk of sampling bias; however, partly controlling for this was a comparison of baseline demographic and clinical characteristics between the study population and patients lost to follow-up that did not show many differences. Unfortunately, high attrition rates and missing data are common challenges encountered in palliative care research [17]. The present study was particularly prone to these problems, as patients lived less than three months following radiotherapy.

In summary, despite their short survival times, patients reported pain relief following radiotherapy, and responders reported superior functional abilities compared to non-responders. Patients with estimated survival times of three months who suffer from painful bone metastases should be considered for palliative radiotherapy, as they may derive not only pain relief from treatment, but also improved functional abilities prior to passing away.
